# Diabetes Mellitus and Thoracic Aortic Disease: Are People With Diabetes Mellitus Protected From Acute Aortic Dissection?

**DOI:** 10.1161/JAHA.112.001404

**Published:** 2012-06-22

**Authors:** Christoph A. Nienaber

**Affiliations:** Heart Center Rostock, Department of Internal Medicine I, Divisions of Cardiology, Pulmonology, and Intensive Care Medicine, University Hospital RostockGermany

**Keywords:** editorials, aortic aneurysm, thoracic, diabetes mellitus, statistics, clinical coding

Are people with diabetes mellitus at reduced risk of acute aortic dissection? It would be a sweet surprise, but the provocative question relates to the findings of a nationwide case–control study by the Center for Clinical Research and Evidence-Based Medicine at the University of Texas in Houston, published in this issue of the *Journal of the American Heart Association* (*JAHA),* ^1^ indicating that diabetes mellitus confers a reduced risk of thoracic aortic aneurysms and dissections. If such inverse relationship is real, this observation would have an important impact and would be revolutionary in the sense that people with diabetes mellitus, despite their chronic disease condition (as bad as it may be), would face a lower risk of suffering and dying from an acute aortic catastrophe. The expected tsunami-like global epidemic of newly diagnosed cases of diabetes mellitus in both Western societies and developing countries^2–5^ would eventually wash ashore some treasures, such as a lower attrition rate of thoracic aortic disease. How wonderful—but is this real?

Crossing medicine and epidemiology obviously produces unexpected results. But let's not get cynical; rather, let's dig deeper into the details of the work of Prakash et al.^1^ The authors used the Nationwide Inpatient Sample (NIS; from the Healthcare Cost and Utilization Project) from 2006 and 2007, which is supposed to approximate a 20% sample of all nonfederal, short-term general and specialty hospitals in the United States, in an attempt to extract discharge-level information on primary and secondary diagnoses and procedures from the *International Classification of Diseases, Ninth Revision, Clinical Modification (ICD-9-CM)* codes and Current Procedural Terminology labels.^1^ Although the NIS may be considered a highly reliable source of data, its data quality is heavily dependent on human factors in coding and weighing diagnoses. The reported inverse relation of diabetes mellitus and acute aortic diseases was derived from cross-tabulation of *ICD-9-CM* codes with NIS diagnoses. In fact, on the basis of just 5425 case records with the primary diagnosis of thoracic aortic aneurysm and dissection (TAAD) (after exclusion of several other diagnostic codes), the authors claim that the prevalence of diabetes mellitus revealed the most consistent disparity between TAAD patients and controls: 9.7 cases of TAAD per 10 000 patients with diabetes mellitus versus 15.6 cases of TAAD per 10 000 discharged patients overall. In other words, patients with TAAD (both aneurysm and dissection) are 40% less likely to have type 2 diabetes mellitus!

Some issues need to be discussed in this context. First, how reliable is a statistical exercise in extracting data from an incomplete data set such as the NIS? Although the authors successfully reproduced their findings in various subgroup association statistics, the source of the input data remained the same. Second, is it fair to use a relatively small sample from NIS data to draw such a far-reaching conclusion as the inverse association of diabetes mellitus and aortic diseases? Remember, the rates were 9.7 per 10 000 in patients with diabetes mellitus and 15.6 per 10 000 in patients without diabetes mellitus. Third, what are the chances that diabetes mellitus was underreported^6^ if the primary diagnosis was an acute aortic problem that dominated the hospitalization, discharge, and aftercare? In such a scenario, a chronic, underlying diabetic condition may not even be mentioned as a secondary diagnosis. Conversely, because the calculations are derived from only hospitalized inpatients (rather than from a population-based sample), patients with diabetes mellitus are likely to be overrepresented in the sample. The magnitude of both effects is, of course, unknown.

Fourth, is it valid and helpful to use hospital discharge information for cross-sectional association studies on the prevalence of certain disease conditions to derive a serious hypothesis? Such administrative input information was never intended to serve any scientific purpose and may not withstand the scrutiny of science. Moreover, no other reliable population-based or hospitalization-derived study documented any kind of association between type 2 diabetes mellitus and aortic diseases. In fact, clear evidence indicates that the incidence of both diabetes mellitus and aortic disease has been rising in recent years in both sexes,^2,5^ and whereas rates of diabetes mellitus continue to rise particularly in younger patients, aortic diseases typically become clinically apparent later in life, beyond the age of 60 years. Again, such disparity in the onset of 2 different disease conditions may contribute to the apparent underrepresentation of diabetes mellitus in the older set of patients with aortic diseases.^5^

Finally, wouldn't it be more convincing to back any daring technical association study with pathophysiology or with a plausible interaction to explain any suggested association of diseases?^7,8^ Or—even better—to find a common denominator for both conditions? Macrophages, which produce proinflammatory cytokines, and matrix-digesting metalloproteinases (MMPs) may play a key role in such an interaction because in aneurysmatic disease those cells operate in all layers of the aorta^9–11^ and promote vessel remodeling and expansion by depositing extracellular matrix degradation products and collagen.^12^ With enhanced glycation (as in diabetes mellitus), more glycated monomeric collagen may be formed, with effects on MMP secretion and marked reduction of MMP-9, MMP-2, and interleukin-6 secretion by activated monocytes, thereby inducing cross-links likely to increase matrix stiffness.^13,14^ The additional observation that glycated extracellular matrix inhibits monocyte MMP production may conceptually explain the potential protection of diabetes mellitus against aortic growth and aneurysm formation.^15^ Nonetheless, mural degradation and expansion seem to work differently in the thoracic and abdominal aorta, and their typical genetic susceptibility loci do not overlap; thus, a mitigating role of hyperglycemia is complex and not fully understood. In fact, the authors quote recent clinical evidence for decreased secretion of metalloproteinase from inflammatory cells in the aortic wall, a process that theoretically inhibits progression of TAAD by stabilizing the extracellular matrix.^16^ Given the assumption that such interaction works in humans, the association with a lower rate of admission or discharge for TAAD is not necessarily a logical consequence. Similarly, single cases of mutations in the smooth muscle cell–specific myosin heavy chain gene *(MYH11)* with upregulation of insulin-like growth factor (and antidiabetic action) are unlikely to explain any association based on hospitalization codes.^17^ Surprisingly, in a study from the same group, Guo et al^18^ recently reported an association of mutations in the smooth muscle α-actin *(ACTA2)* gene with thoracic aortic disease, stroke, and coronary artery disease, with the latter being clearly and causally related to type 2 diabetes mellitus.

What does the study by Prakash et al^1^ tell us? Well, the hypothetical association presented here is certainly intriguing but should be viewed as a hypothesis-generating observation that suggests the need for further effort to either confirm or refute a causal relationship between diabetes mellitus and mitigated evolution of aortic aneurysm formation or prevention of rupture.
